# Research and Development of Advanced Computing Technologies

**DOI:** 10.1155/2015/239723

**Published:** 2015-03-26

**Authors:** Shifei Ding, Zhongzhi Shi, Ahmad Taher Azar

**Affiliations:** ^1^School of Computer Science and Technology, China University of Mining and Technology, Xuzhou 221116, China; ^2^Key Laboratory of Intelligent Information Processing, Institute of Computing Technology, Chinese Academy of Sciences, Beijing 100190, China; ^3^Faculty of Computers and Information, Benha University, Benha 13511, Egypt

Welcome to this special issue. Advanced computing technologies are one of the hot research fields in artificial intelligence. This special issue aims to promote the research, development, and applications of advanced computing technologies by providing a high-level international forum for researchers and practitioners to exchange research results and share development experiences. The papers in this edition were selected among the highest rated papers in submitted manuscripts. The selection of papers featured here covers the topics of the main advanced computing technologies and experimental studies of some application systems.

C.-W. Lin et al. proposed a privacy-preserving data mining method by using a compact prelarge GA-based algorithm to delete transactions for hiding sensitive itemsets. This method solves the limitations of the evolutionary process by adopting both the compact GA-based mechanism and the prelarge concept. A. Adam et al. build a framework for peak detection on EEG signals in time domain analysis using feature selection and classifier parameters estimation based on particle swarm optimization. The proposed framework tries to find the best combination for all the available features that offers good peak detection and a high classification rate from the results in the conducted experiments. Y. Pei et al. propose a method to establish a human model in high dimensional search space by kernel classification for enhancing interactive evolutionary computation search. N. Sulaiman et al. propose a modified artificial bee colony algorithm to enhance the convergence speed and improve the ability of the standard artificial bee colony algorithm to reach the global optimum by balancing the exploration and exploitation processes. This method was tested on the reactive power optimization problem and has outperformed other compared algorithms. Aiming at solving the biased feature selection in random forests, T. T. Nguyen et al. propose a modified random forests algorithm to select good features in learning random forests for high dimensional data. L. Li et al. put forward a prediction model based on membrane computing optimization algorithm for chaos time series; the model optimizes simultaneously the parameters of phase space reconstruction and least squares support vector machine by using membrane computing optimization algorithm. M. R. Al-Othman et al. propose a heuristic ranking approach on capacity benefit margin (CBM) determination using Pareto-based evolutionary programming technique. This paper introduces a novel multiobjective approach for CBM assessment taking into account tie-line reliability of interconnected systems and presents a new Pareto-based evolutionary programming (EP) technique used to perform a simultaneous determination of CBM for all areas of the interconnected system.

In order to build a QoS-aware bufferless network-on-chip scheme for datacenters, J. Fang et al. propose QBLESS, a QoS-aware bufferless NoC scheme for datacenters. QBLESS consists of two components: a routing mechanism (QBLESS-R) that can substantially reduce flit deflection for high-priority applications and a congestion-control mechanism (QBLESS-CC) that guarantees performance for high-priority applications and improves overall system throughput. S. K. Chandrasekaran et al. propose a framework of primary path reservation admission control protocol, which achieves improved QoS by making use of backup route combined with resource reservation. In this paper, a network topology has been simulated and the present approach proves to be a mechanism that admits the session effectively. V. Uc-Cetina et al. propose a Markov decision process model for solving the web service composition problem. Iterative policy evaluation, value iteration, and policy iteration algorithms are used to experimentally validate the present approach. S. U. Rehman and A. Nadeem propose a novel approach to the testing of multiagent systems based on Prometheus design artifacts. In the proposed approach, different interactions between the agent and actors are considered to test the multiagent system. M. Bal et al. study 11 machine learning methods (SVM, MLP, C4.5, etc.) for the inference mechanism of medical decision support system based on ALARM network. The performances of 11 machine learning algorithms are tested on 44 synthetic data sets (11 different dependent variables and 4 different dataset sizes). The comparison of algorithms applied two different tests (statistically difference and average rank tests). C4.5 decision tree is the best algorithm according to both of the tests for our 44 datasets. The datasets having more samples can be better predicted than having fewer samples.

A comparative study on interaction of form and motion processing streams by applying two different classifiers in mechanism for recognition of biological movement is proposed by B. Yousefi and C. K. Loo. The presented approach has addressed a very substantial interrelevant comparison of the interaction of two processing streams of mammalian brain visual system. For mining data streams, A. O. Díaz et al. present a fast adapting ensemble method which adapts very quickly to both abrupt and gradual concept drifts, and the method has been specifically designed to deal with recurring concepts. After initializing color image by utilizing the unsupervised Orchard and Bouman clustering technique, D. Khattab et al. present a comparative study using different color spaces to evaluate the performance of color image segmentation using the automatic GrabCut technique. M.-L. Huang et al. combine feature selection and SVM recursive feature elimination to investigate the classification accuracy of multiclass problems for Dermatology and Zoo databases. Meanwhile, Taguchi's method was jointly combined with SVM classifier in order to optimize parameters to increase classification accuracy for multiclass classification.

For image compression, N. A. Abu and F. Ernawan propose a psychovisual threshold on the large discrete cosine transform (DCT) image block which will be used to automatically generate the much needed quantization tables. G. C. Kim proposes a fully autonomous feature selection and camouflaged object detection method based on the online analysis of spectral and spatial features.

Z. Wang et al. point out that spectral clustering algorithms applied in community detection have two inadequacies and present a novel community detection algorithm based on topology potential and spectral clustering that contains rich structural information of the network. A. Rodan et al. utilize an ensemble of multilayer perceptrons whose training is obtained using negative correlation learning for predicting customer churn in a telecommunication company. S. Chen et al. describe a hybrid approach for forecasting fraudulent financial statements. The authors firstly screen the important variables using the stepwise regression and then use logistic regression, support vector machine, and decision tree to construct the classification models to make a comparison.

J. D. Gazzano et al. propose a complete grid infrastructure for distributed high-performance computing based on dynamically reconfigurable FPGAs. This infrastructure was tested and significant performance gains have been achieved. P systems are a class of distributed parallel computing models, H. Peng et al. present a novel clustering algorithm, which is inspired from mechanism of a tissue-like P system with a loop structure of cells, called membrane clustering algorithm. Q. Luo et al. propose a discrete bat algorithm (DBA) for optimal problem of permutation flow shop scheduling. The authors construct a direct relationship between the job sequence and the vector of individuals in DBA.

